# Periodontitis in patients with psoriasis: A systematic review and meta‐analysis

**DOI:** 10.1111/odi.13617

**Published:** 2020-09-18

**Authors:** Xinze Zhang, Hongqiu Gu, Shang Xie, Yingying Su

**Affiliations:** ^1^ Department of Stomatology Beijing Tiantan Hospital Capital Medical University Beijing China; ^2^ China National Clinical Research Center for Neurological Diseases Beijing Tiantan Hospital Capital Medical University Beijing China; ^3^ Department of Oral and Maxillofacial Surgery Peking University School and Hospital of Stomatology Beijing China

**Keywords:** periodontitis, psoriasis, systematic review

## Abstract

**Objective:**

The present study aimed to summarize and update the evidence regarding the association between periodontitis and psoriasis.

**Methods:**

The present systematic review was conducted under the guidelines of Transparent Reporting of Systematic Reviews and Meta‐Analyses (PRISMA) and was recorded in the PROSPERO database, under registration number CRD42017063799. Three databases (MEDLINE, Embase, and Cochrane Library) were searched up to March 2020. Case–control or cohort studies assessing the association between periodontitis and psoriasis were identified. Quantitative synthesis was conducted with meta‐analysis.

**Results:**

A total of 13 studies (11 case–control and two cohort studies) assessing the association between periodontitis and psoriasis were included. Of these 13 articles, 9 showed the prevalence of periodontitis or psoriasis. Therefore, meta‐analyses were conducted with data retrieved from the nine studies included. Pooled effect estimate for nine studies showed that patients with periodontitis associated with a higher risk of psoriasis with a pooled OR of 2.87 (95% CI, 1.75–4.69).

**Conclusions:**

This systematic review demonstrated a positive association between periodontitis and psoriasis; however, a causal relationship cannot be established. Due to the weak evidence, caution should be taken when interpreting the results regarding periodontal parameters. Well‐designed prospective studies are necessary to evaluate interactions between both diseases.

## 1 INTRODUCTION

Periodontitis is a chronic inflammatory disease triggered by bacterial infections that activate the host's immune response, thereby causing destruction of the supporting tissues of the teeth, forming periodontal pockets, and eventually leading to tooth loss (Kinane & Bartold, 2007). It is one of the most common oral diseases that affects approximately 20%–60% of the world population (Albandar, 2002; Corbet, Zee, & Lo, 2002; Gjermo, Rosing, Susin, & Oppermann, 2002; Sheiham & Netuveli, 2002). Periodontitis is seen as resulting from a complex interplay of the oral microbial community with the host response, modified by genetic and environmental factors. In recent decades, there has been increasing evidence supporting a strong association between periodontitis and systemic conditions, including cardiovascular diseases (Liccardo et al., 2019), metabolic syndrome (Tegelberg et al., 2019), rheumatoid arthritis (Lucchino et al., 2019), and adverse pregnancy outcomes (Bassani, Olinto, & Kreiger, 2007).

Psoriasis is a systemic inflammatory disorder manifesting in the skin or joints or both, with an estimated global prevalence ranging from 0.5% to 4.6% (Lebwohl, 2003). Although the pathogenesis of psoriasis is still not fully understood, the immune system is now recognized as having a prominent role in the development of this disease (Nickoloff, Qin, & Nestle, 2007). Psoriatic skin lesions originate as a result of dysregulated interactions of the host immune system with resident cutaneous cell types. Psoriasis has been reported to be associated with multiple comorbidities, including cardiovascular disease (Armstrong, Harskamp, & Armstrong, 2013), metabolic syndrome (Armstrong, Harskamp, & Armstrong, 2013b), diabetes (Armstrong, Harskamp, & Armstrong, 2013c), and hypertension (Armstrong, Harskamp & Armstrong 2013a).

Since psoriasis and periodontitis have similar pathogenic mechanisms and associated conditions in common, there has been a renewed interest in the research of possible links between psoriasis and periodontitis. The current hypotheses of common etiopathogenesis pathways between psoriasis and periodontitis involve several possible mechanisms such as an amplified inflammatory reaction and T‐cell activation (Pischon et al., 2008) and lower concentration of salivary IgA and Iysozyme (Ganzetti et al., 2014); this may result in an increased inflammatory response. Other hypotheses indicated specific characteristics of the salivary microbiota and reduced levels of neutrophil gelatinase‐associated lipocalin and transferrin in saliva of patients with psoriasis, which might result in an abnormal host response to the oral microbiota and impact oral homeostasis (Belstrom et al., 2020). Several clinical studies reported increased alveolar bone loss, increased severity of periodontal disease, and a higher number of missing teeth in patients with psoriasis compared with match controls (Egeberg, Mallbris, Gislason, Hansen, & Mrowietz, 2017; Preus, Khanifam, Kolltveit, Mork, & Gjermo, 2010; Su, Huang, Hu, Yu, & Chang, 2017), while a few studies showed no significant relationship (Fadel et al., 2013; Sezer et al., 2016). A systematic review and meta‐analysis published in 2017 evaluating five observational studies (2 cohort and 3 case–control studies) reported that patients with periodontitis presented a significantly elevated risk for psoriasis (pooled RR = 1.55, 95% CI: 1.35–1.77; *p* < .001; Ungprasert, Wijarnpreecha, & Wetter, 2017). This review was built on limited evidence, and the influence of insecure periodontitis diagnostic criteria was not taken into consideration when performing data synthesis. Therefore, the present review aimed to update the meta‐analysis with additional studies published through to March 2020 to provide further evidence about the association between periodontitis and the risk of psoriasis.

## 2 MATERIALS AND METHODS

### 2.1 Protocol and registration

The present systematic review was recorded in the PROSPERO database, under registration number CRD42017063799, and was conducted under the PRISMA guidelines (Liberati et al., 2009).

### 2.2 Study design and eligibility criteria

This review aimed to answer the following question: Is there an association between periodontitis and psoriasis demonstrated in observational studies? The PECO strategy for the question was as follows: in patients diagnosed with psoriasis and/or periodontitis (P), the exposure was the presence of periodontitis and/or psoriasis (E), compared with patients with no periodontitis and/or no psoriasis (C). The outcome was any measure of the prevalence of psoriasis and periodontitis or other forms of periodontal evaluation (O).

The included studies had to meet the following criteria: case–control, cross‐sectional, or cohort (retrospective or prospective) design; carried out in adult subjects; report the prevalence of psoriasis or periodontitis. We excluded studies lacking raw data after contacting the authors for complete data, as well as review articles, case reports, and articles without the results of interest.

### 2.3 Search strategy

Articles published before March 2020 were systematically searched through MEDLINE, Embase, and the Cochrane Library. References of included articles and related review articles were manually searched. The following keywords and MeSH terms were used: periodontitis, periodontal diseases, periodont*, periodontics, periodontitis [MeSH Terms], periodontal diseases [MeSH Terms], psoriasis, pityriasis, pustular psoriasis, psoria*, psoriasis [MeSH Terms], and pityriasis [MeSH Terms]. The asterisk symbol (*) was used as truncation.

Eligible articles were independently identified by two reviewers (XZZ and YYS) for possible inclusion in the systematic review and meta‐analysis. Full texts were obtained and evaluated independently for articles appearing to meet the inclusion criteria and for those lacking sufficient information in their title and abstract to allow clear decisions. Disagreements were resolved by discussion.

### 2.4 Data extraction and data synthesis

Data were extracted independently by two reviewers (XZZ and YYS) and included study identifier, study design, number of participants per group, outcome measures, and diagnosis of periodontitis and psoriasis. Disagreements were settled with discussion, or by a third author (SX). For binary outcomes, we first extracted the multivariable‐adjusted effect measurements of odds ratio (OR), relative risk (RR), or hazard ratio (HR) and their 95% confidence intervals, if these were not available, we would extract crude effective measurements or use estimated measure based on data reported.

### 2.5 Quality assessment

The quality assessment for observational studies included in present review was based on the Newcastle–Ottawa Scale (NOS) tool evaluating the following criteria: adequacy of case definition (periodontitis/psoriasis); representativeness of the cases; selection and definition of controls; comparability of cases and controls with respect to confounding factors; assessment of outcome (periodontitis/psoriasis).

### 2.6 Statistical analysis

Cochrane test was used to assess heterogeneity across studies. Also, we calculated the *I* ^2^ statistic to measure the consistency between trials with values of 25%, 50%, and 75% representing low, moderate, and high degrees of heterogeneity, respectively. Pooled odds ratio (OR) was estimated using fixed effects models when there was low heterogeneity or moderate heterogeneity (*I* ^2^ < 50%), and randomized effects models when there was more than moderate heterogeneity (*I* ^2^ ≥ 50%). Forest plots were generated for graphical presentations of outcomes. All *p*‐values were two‐tailed with the statistical significance set at <.05. All analyses were conducted using R software (R version 3.6.1).

### 2.7 Sensitivity analyses

To test for the robustness of the results, several sensitivity analyses were performed. Subgroup analysis results were reported according to the study type (case–control or cohort). We also considered subgroup analysis by different levels of diagnostic criteria. Descriptive analyses were conducted about parameters involving in the assessment of periodontitis, such as remaining teeth, missing teeth, probing depth (PD), and clinical attachment level (CAL).

## 3 RESULTS

### 3.1 Study selection and characteristics

**Figure 1:**
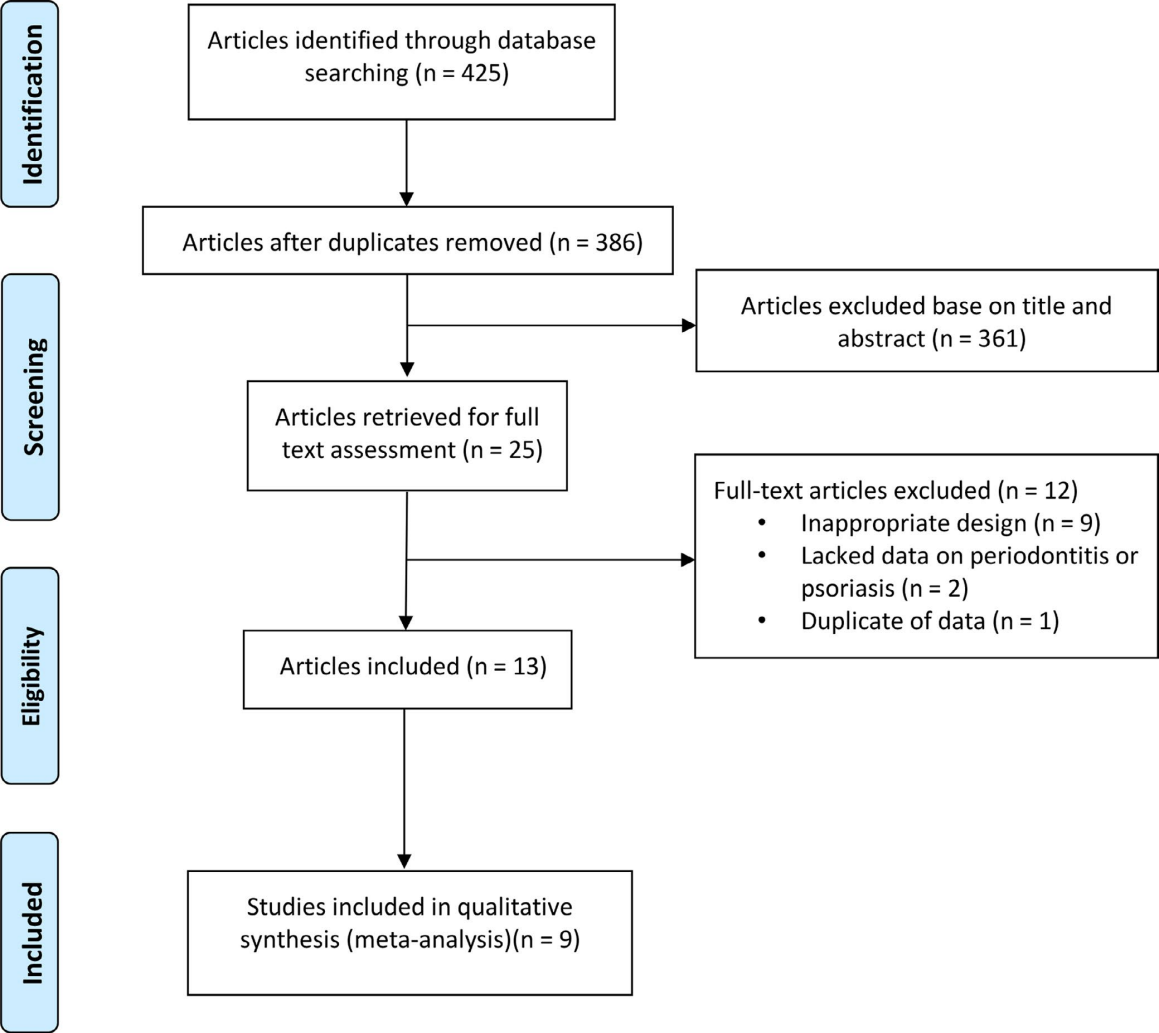
Flow diagram of the searching processes and results based on PRISMA guidelines

Among the 425 potentially relevant publications identified in searching databases, 39 duplicates were removed. After reading titles and abstracts, 361 publications were excluded, resulting in 25 studies for full‐text evaluation. A total of 13 studies met the eligibility criteria and were finally included in this systematic review (Table 1). The articles excluded from the analysis, and the reasons for their exclusion, are shown in Figure 1. The included articles were 11 case–control (Fadel et al., 2013; Ganzetti et al., 2015; Lazaridou et al., 2013; Mendes, Cota, Costa, Oliveira, & Costa, 2019; Painsi, Hirtenfelder, Lange‐Asschenfeldt, Quehenberger, & Wolf, 2017; Preus et al., 2010; Sarac, Kapicioglu, Cayli, Altas, & Yologlu, 2017; Sharma, Raman, & Pradeep, 2015; Skudutyte‐Rysstad, Slevolden, Hansen, Sandvik, & Preus, 2014; Woeste, Graetz, Gerdes, & Mrowietz, 2019) and 2 cohort studies (Keller & Lin, 2012; Nakib, Han, Li, Joshipura, & Qureshi, 2013) published in the English language between 2010 and 2019 and conducted in 13 different countries and regions across Asia, Europe, and America. Of these 13 articles, 9 showed prevalence of periodontitis or psoriasis (Antal, Braunitzer, Mattheos, Gyulai, & Nagy, 2014; Ganzetti et al., 2015; Keller & Lin, 2012; Lazaridou et al., 2013; Mendes et al., 2019; Nakib et al., 2013; Painsi et al., 2017; Preus et al., 2010; Skudutyte‐Rysstad et al., 2014), five reported data for continuous variables, including PD (Mendes et al., 2019; Sharma et al., 2015), clinical attachment level (CAL) (Mendes et al., 2019; Sharma et al., 2015), number of missing teeth (Skudutyte‐Rysstad et al., 2014; Woeste et al., 2019), or number of remaining teeth (Fadel et al., 2013; Mendes et al., 2019).

**Table 1:** Characteristic of included studies

Study	Study design	Sample size of cases (male/female)	Sample size of comparison subjects (male/female)	Periodontitis diagnosis	Psoriasis diagnosis	Outcome	Outcome measurements
Preus et al. (2010), Norway	Case–control	155 patients with psoriasis (43.2%/56.8%)	155 age‐ and gender‐matched controls (43.2%/56.8%)	Bone level determined by bite‐wing radiographs. No further information about the definition of periodontitis	Self‐reported physician‐diagnosed psoriasis though health questionnaires	Periodontitis	Percentage of the individuals with lower bone level
							78% (psoriasis) versus 17% (control)
Keller et al. (2012), Taiwan	Retrospective cohort study	115,365 patients with chronic periodontitis (47.9%/52.1%)	Randomly selected 115,365 comparison patients to match the study patients (47.9%/52.1%)	Cases of periodontitis were identified from the Longitudinal Health Insurance Database. Subjects have at least 2 diagnostic codes of periodontitis in the database	Presence of diagnostic code for psoriasis was used to identify the diagnosis of psoriasis. No further verification was made	Psoriasis	After censoring participants who died during the follow‐up period, and adjusting for monthly income and geographical region, compared with comparison patients, the hazard ratio (HR) of psoriasis for patients with chronic periodontitis was 1.52 (95% CI 1.38–1.70)
Lazaridou et al. (2012), Greece	Case–control	100 patients with chronic plaque psoriasis (43%/57%)	100 age‐ and gender‐matched controls (43%/57%)	Community Periodontal Index (CPI) score, CPI ≥1 was defined as periodotitis	Psoriasis Area and Severity Index (PASI) score	Periodontitis	Significant correlation of psoriasis and periodontitis was found (OR: 2.486, 95% CI: 1.002–5.842, *p* = .049)
Fadel et al. (2013) Sweden	Case–control	89 patients with mild‐ to‐moderate chronic plaque psoriasis (52%/48%)	54 people without psoriasis (39%/61%)	Mild periodontitis: radiographic alveolar bone level of 2–3.5 mm from CEJ and BOP; moderate periodontitis: radiographic alveolar bone level of 4–5.5 mm from CEJ and BOP; severe periodontitis: radiographic alveolar bone level of ≥ 6 mm from CEJ and BOP	No description	Periodontitis	Percentage of the individuals with moderate‐to‐severe periodontitis
							24% (psoriasis) versus 13% (control)
Nakib et al. (2013), USA	Prospective cohort study	11,358 cases of periodontitis (all are female)	70,020 subjects in Nurses Health Study cohort who did not have psoriasis and periodontitis	Self‐reported	Self‐reported physician‐diagnosed psoriasis	Psoriasis	After adjusting for age, BMI, smoking status, and intensity, alcohol intake, physical activity, and number of teeth, the relative risk (RR) of patients with periodotitis was 1.40 (95% CI 1.13, 1.73)
Antal et al. (2014), Hangary	Case–control	82 psoriasis patients (45%/55%)	89 age‐matched controls (51%/49%)	Healthy: no clinical attachment loss (CAL) or BOP (CPI = 1). Early periodontitis:CAL ≥ 1 mm in ≥ 2 teeth (CPI = 2). Moderate periodontitis: 3 sites with CAL ≥ 4 mm and at least 2 sites with probing depth (PD) ≥3 mm (CPI = 3). Severe periodontitis: CAL ≥ 6 mm in ≥ 2 teeth and PD ≥ 5 mm in ≥ 1 site (CPI = 4)	Participants were selected from the patients of the Psoriasis Outpatient Unit of the Department of Dermatology and Allergology, University of Szeged. Psoriasis was defined as ICD‐10 L40.0‐L40.9	Periodontitis	Percentage of the individuals with periodontitis
							72/82 (psoriasis) versus 54/89 (control)
Ganzetti et al. (2014), Italy	Case–control	60 patients with psoriasis (42%/58%)	45 healthy controls (47%/53%)	Chronic periodontitis was considered localized (≤30% of sites involved) or generalized (>30%) clinical attachment loss (CAL). Severity was characterized by the amount of CAL (Slight = 1–2 mm, Moderate = 3–4 mm, Severe ≥ 5 mm)	No description	Periodontitis	Percentage of the individuals with periodontitis
							23/60 (psoriasis) versus 9/45 (control)
Sharma et al. (2014), India	Case–control	33 patients with psoriasis (57.6%/42.4%)	35 systemically healthy subjects (51.4%/48.6%)	Subjects with ≥2 interproximal sites with PD ≥5 mm or ≥2 interproximal sites with periodontal attachment loss (PAL) ≥4 mm with radiographic evidence of bone loss (distance of Cemento‐Enamel Junction to alveolar bone crest of 6 mm or more) were defined as chronic periodontitis	PASI score	Periodontitis	PD and Periodontal Attatchment Loss (PAL) were significantly greater in psoriasis group compared with healthy subjects. The number of missing teeth was significantly higher among psoriasis group. Logistic analysis showed significant relationship between PD and severity of psoriasis and between PAL and severity of psoriasis
Skudutyte‐Rysstad et al. (2014), Norway	Case–control	50 patients with psoriasis (24%/76%)	121 controls (50%/50%)	Periodontitis was defined according to the case definitions for surveillance of periodontitis proposed by the Centers for Disease Control and Prevention (CDC) and the American Academy of Periodontology (AAP)	PASI score	Periodontitis	Compared with controls, psoriasis individuals had significantly more missing teeth and more sites with plaque and bleeding on probing. The prevalence of moderate and severe periodontitis was significantly higher among psoriasis individuals (24%) compared with healthy controls (10%). Similarly, 36% of psoriasis cases had one or more sites with radiographic bone loss ≥3 mm, compared with 13% of controls. Psoriasis was significantly associated with moderate/severe periodontitis and radiographic bone loss
Sarac et al. (2016), Turkey	Case–control	76 psoriatic patients (40.8%/59.3%)	76 people without any systemic disease (31.6%/68.4%)	CPI score: 0: no periodontal disease, 1: gingival bleeding, 2: calculus detected while probing, 3: the depth of pocket 4–5 mm, 4: the depth of pocket 6 mm and above	PASI score	Periodontitis	Community Periodontal Index (CPI) scores of the patients were significantly higher than those of the control groups (*p* = .01)
Painsi et al. (2017), Austria	Retrospective case–control	209 psoriasis patients (61.2%/38.8%)	91 chronic spontaneous urticarial (CSU) patients (40.7%/59.3%)	No description	PASI score	Periodontitis	The prevalence of periodontitis was significantly increased in psoriasis compared to CSU patients with an OR of 3.76 (95% confidence interval, CI = 1.60–10.27, *p* = .001)
Mendes et al. (2019), Brazil	Case–control	397 patients with psoriasis (40.1%/59.9%)	359 people without any dermatological disease (37.3%/62.7%)	Using CDC/AAP definitions. Severe periodontitis was defined as having two or more interproximal sites with ≥CAL 6 mm (not on the same tooth) and one or more interproximal site(s) with PD ≥ 5 mm. Moderate periodontitis, defined as two or more interproximal sites with CAL ≥ 4 mm (not on the same tooth) or two or more interproximal sites with PD ≥ 5 mm, also not on the same tooth; and mild periodontitis, defined as ≥2 interproximal sites with CAL ≥3 mm and ≥2 interproximal sites with PD ≥4 mm (not on the same tooth) or 1 site with PD ≥5 mm	PASI score	Periodontitis	Individuals with psoriasis presented a chance 1.72 higher of having periodontitis than controls (OR = 1.72; 95% CI 1.28–2.32; *p* < .001) in univariate analysis. There was no significant independent association between psoriasis and periodontitis in the multivariate model (OR = 1.27; 95% CI 0.89 –1.83; *p* = .188)
Woeste et al. (2019), Germany	Case–control	100 psoriasis patients (59%/41%)	101 non‐psoriasis control individuals (42.6%/57.4%)	CPI score: 1 or 2: periodontally healthy; 3 or 4: presence of gingival or periodontal pockets with probing depth of 4 mm or greater	PASI score	Periodontitis	Psoriasis group had significantly higher values on Bleeding on Probing (BOP) and the Community Periodontal Index (CPI) compared with matched control individuals

With regard to the definition criteria for PD, two studies (Mendes et al., 2019; Skudutyte‐Rysstad et al., 2014) used Centers for Disease Control and Prevention (CDC) and the American Academy of Periodontology (AAP) definition; three (Lazaridou et al., 2013; Sarac et al., 2017; Woeste et al., 2019) used the Community Periodontal Index (CPI); three (Fadel et al., 2013; Preus et al., 2010; Sharma et al., 2015) defined periodontitis by the evidence of radiographic alveolar bone loss; two (Antal et al., 2014; Ganzetti et al., 2015) reported CAL and/or PD; one (Keller & Lin, 2012) identified cases of periodontitis from the Longitudinal Health Insurance Database by diagnostic codes; one (Nakib et al., 2013) defined periodontitis according to self‐reported history of periodontal bone loss; and one (Painsi et al., 2017) did not describe the diagnosis criteria of periodontitis. The diagnosis of psoriasis in seven studies (Lazaridou et al., 2013; Mendes et al., 2019; Painsi et al., 2017; Sarac et al., 2017; Sharma et al., 2015; Skudutyte‐Rysstad et al., 2014; Woeste et al., 2019) was based on Psoriasis Area and Severity Index (PASI) score. In two studies, psoriasis was self‐reported (Nakib et al., 2013; Preus et al., 2010). Two reported the presence of diagnostic code for psoriasis (Antal et al., 2014; Keller & Lin, 2012). And one had no description about the diagnosis of psoriasis (Fadel et al., 2013). Tables 2 and 3 showed the quality assessment of the included case–control and cohort studies, respectively. Based on the Newcastle–Ottawa Scale, 9 studies (Fadel et al., 2013; Keller & Lin, 2012; Lazaridou et al., 2013; Mendes et al., 2019; Nakib et al., 2013; Sarac et al., 2017; Sharma et al., 2015; Skudutyte‐Rysstad et al., 2014; Woeste et al., 2019) were rated as a total score of > 5, and 4 studies (Antal et al., 2014; Ganzetti et al., 2015; Painsi et al., 2017; Preus et al., 2010) as a score of ≤ 5, indicating a high risk of bias.

**Table 2:** Quality assessment of included case–control studies according to New Castle–Otawa Scale (NOS)

Study	Selection				Comparability	Exposure			Total score
	Adequate definition of cases	Representativeness of the cases	Seletion of controls	Definition of controls	Comparability of cases and controls on the basis of the design or analysis	Ascertainment of exposure	Same method of ascertainment for cases and controls	Non‐response rate	
Preus et al. (2010), Norway				*	*	*	*	*	5
Lazaridou et al. (2012), Greece	*	*		*	*	*	*	*	7
Fadel et al. (2013), Sweden		*	*	*	*	*	*	*	7
Antal et al. (2014), Hangary				*	*	*	*	*	4
Ganzetti et al. (2014), Italy				*	*	*	*	*	5
Sharma et al. (2014), India	*			*	*	*	*	*	6
Skudutyte‐Rysstad et al. (2014), Norway	*			*	*	*	*	*	6
Sarac et al. (2016), Turkey	*			*	*	*	*	*	6
Painsi et al. (2017), Austria	*			*	*			*	4
Mendes et al. (2019), Brazil	*		*	*	*	*	*	*	7
Woeste et al. (2019), Germany	*			*	*	*	*	*	6

Note

* It is used to access the quality of the original studies. A study can be awarded a maximum of one star for each numbered item for case‐control study.

**Table 3:** Quality assessment of included cohort studies according to New Castle–Otawa Scale (NOS)

Study	Selection				Comparability	Outcome			Total score
	Representativeness of the exposed cohort	Seletion of the non exposed cohort	Ascertainment of exposure	Demonstration that outcome of interest was not present at start of study	Comparability of cohorts on the basis of the design or analysis	Assessment of outcome	Was followed up long enough for outcomes to occur	Adequacy of follow‐up of cohorts	
Keller et al. (2012), Taiwan	*	*	*	*	**	*	*	**	10
Nakib et al. (2013), USA		*		*	**		*	**	7

Note

*, **It is are used to rate the quality of the original studies.

### 3.2 Meta‐analysis

**Figure 2:**
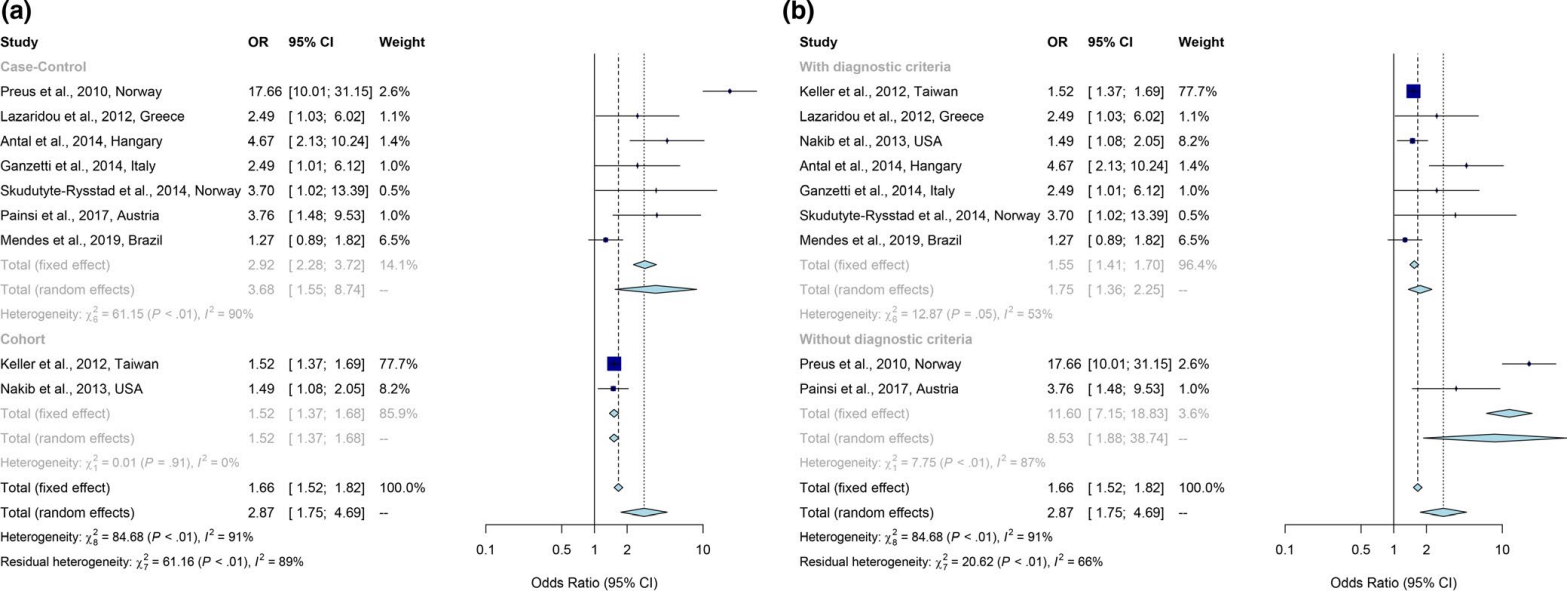
Meta‐analyses of odds ratio for the association between periodontitis and psoriasis according to study design (a) and periodontitis diagnostic criteria (b). Horizontal lines represent 95% confidence intervals (CIs). Diamonds represent the overall odds ratio estimate with its 95% CI

In total, 314,121 participants were included in the main outcome meta‐analysis. Pooled effect estimates for 9 studies (Antal et al., 2014; Ganzetti et al., 2015; Keller & Lin, 2012; Lazaridou et al., 2013; Mendes et al., 2019; Nakib et al., 2013; Painsi et al., 2017; Preus et al., 2010; Skudutyte‐Rysstad et al., 2014) (OR and 95% CI) showed that patients with periodontitis has increased odds for psoriasis with a pooled OR of 2.87 (95% CI, 1.75–4.69; Figure 2a). However, statistical heterogeneity was considerable (*I* ^2^ = 91%) (Figure 2a). When we stratified by study design, the heterogeneity subsided for cohort studies (*I* ^2^ = 0%), while there was still relatively substantial heterogeneity for the case–control studies (*I* ^2^ = 90%). But case–control studies estimated an association with an increased magnitude (OR = 3.68; 95% CI, 1.55–8.74), whereas cohort studies showed a decreased magnitude (OR = 1.52; 95% CI, 1.37–1.68) (Figure 2a). Stratification by with or without restrictive diagnosis criteria for periodontitis provided further insight: heterogeneity for studies with secure PD diagnosis was relatively low (*I* ^2^ = 53%) and showed a pooled OR of 1.75 (95% CI, 1.36–2.25; Figure 2b), while studies with insecure PD diagnosis harbor high heterogeneity (*I* ^2^ = 87%) with a pooled OR of 2.87 (95% CI, 1.75–4.69; Figure 2b).

### 3.3 Analyses of periodontal clinical parameters

In addition to the association between periodontitis and psoriasis, parameters involving in the assessment of periodontitis were also analyzed in our systematic review. Since the number of studies was too small to perform a meta‐analysis, only descriptive analyses were conducted. For the tooth loss analysis, two studies (Fadel et al., 2013; Mendes et al., 2019) reported remaining teeth and another two (Skudutyte‐Rysstad et al., 2014; Woeste et al., 2019) described missing teeth. Fadel et al. reported the mean for remaining teeth was 24 ± 4 for the psoriasis group and 26 ± 3 for the control group. In study by Mendes et al., the mean for the remaining teeth was 23.26 ± 2.9 for the psoriasis group and 24.83 ± 3.02 for the control group. In study conducted by Skudutyte‐Rysstad et al., the mean value of missing teeth was 2.4 ± 3.9 in the psoriasis group and 1.4 ± 2.5 in the non‐psoriasis group. Woeste et al. reported that missing teeth = 2.92 ± 4.88 for the psoriasis group and 1.64 ± 4.16 for the control group. The evaluation of these indexes collectively indicated the vulnerability of psoriasis patients to teeth loss. Two studies (Mendes et al., 2019; Sharma et al., 2015) reported PD and CAL analysis. For PD, the mean value of 3.12 ± 0.86 (mm) was recorded for the psoriasis group in comparison with 2.85 ± 0.58 (mm) of the control group in Mendes's study. Sharma et al. showed that the mean values and deviations of PD were 4.18 ± 0.705 (mm) and 2.79 ± 1.053 (mm) for the psoriasis and control group, respectively. For CAL, the mean values and deviations were 3.59 ± 0.92 (mm) for the psoriasis group and 3.4 ± 0.69 (mm) for the control in Mendes's study. Sharma et al. found mean values of 2.27 ± 0.676 (mm) for the case and 1.33 ± 0.883 (mm) for the control group. Both studies revealed that individuals with psoriasis presented greater PD and CAL compared with control group.

## 4 DISCUSSION

Psoriasis is a chronic inflammatory skin condition that can markedly affect psychological, occupational, and the quality of life of affected individuals. Knowledge about the risk factors/indicators associated with the occurrence/development of psoriasis is crucial. The management of specific risk factors could help to reduce the prevalence of psoriasis. The primary goal of this systematic review was to assess the relationship between periodontitis and psoriasis. The prevalence of periodontitis in individuals with psoriasis is one of the primary components of this relationship. The meta‐analysis was primarily conducted considering all included studies with most adjusted ORs. The overall results suggested a positive association between psoriasis and prevalence of periodontitis. The risk of psoriasis was higher in subjects with periodontitis compared with the control group (OR, 2.87; 95% CI, 1.75–4.69). However, the pooled results were associated with substantial heterogeneity, which may mainly be attributed to variability across the included studies, in terms of study design and periodontitis diagnostic criteria. The quality assessment has shown that the quality of most of the case–control studies was lower than that of the cohort studies. Low methodological quality may contribute to the significant heterogeneity among case–control studies. When case–control and cohort studies were separately input in meta‐analysis, both the heterogeneity and the pooled ORs changed, indicating that heterogeneity of the study designs could modify the magnitude of the association and restrict its interpretation. In addition, since a large number of risk factors are shared between periodontitis and psoriasis, it is essential to evaluate other independent factors that may confound the real association between the two diseases. The majority of the original studies analyzed in the present meta‐analysis have included age and gender (Keller & Lin, 2012; Lazaridou et al., 2013; Mendes et al., 2019; Nakib et al., 2013; Painsi et al., 2017; Skudutyte‐Rysstad et al., 2014). The other adjusted confounding factors were smoking (Antal et al., 2014; Lazaridou et al., 2013; Mendes et al., 2019; Nakib et al., 2013; Skudutyte‐Rysstad et al., 2014), alcohol intake (Mendes et al., 2019; Nakib et al., 2013), body mass index (Nakib et al., 2013; Skudutyte‐Rysstad et al., 2014), medicaments use (Mendes et al., 2019; Skudutyte‐Rysstad et al., 2014), and socioeconomic factors (i.e. education, income, or geographical region) (Keller & Lin, 2012; Skudutyte‐Rysstad et al., 2014). Two studies have not included any confounding factors (Ganzetti et al., 2015; Preus et al., 2010). This may also result in the heterogeneity of the studies.

Another factor affecting the pooled result was periodontitis diagnostic criteria used in the included studies. Periodontal evaluation is a combination of several clinical parameters. PD, CAL, and vertical bone loss are recommended for the definition of periodontitis by the American Academy of Periodontology (AAP) and the European Federation of Periodontology (Papapanou et al., 2018). Besides, CPI is widely used for periodontal assessment in epidemiological studies with large samples, although it is criticized for the validity of the estimates (Oppermann, Haas, Rosing, & Susin, 2015). In this review, included studies were classified as secure or insecure according to the periodontal diagnosis criteria used. Seven studies which defined periodontitis by clinical/radiographic methods were considered as secure (Antal et al., 2014; Ganzetti et al., 2015; Keller & Lin, 2012; Lazaridou et al., 2013; Mendes et al., 2019; Nakib et al., 2013; Skudutyte‐Rysstad et al., 2014); two using self‐reported diagnosis method or no description were classified as insecure (Painsi et al., 2017; Preus et al., 2010). When meta‐analysis was conducted regarding the diagnosis classification (secure/insecure), a decrease in heterogeneity was observed for secure studies, while heterogeneity was still significant for insecure studies, indicating that studies using secure periodontal diagnosis presented more reliable estimates.

Previous systematic reviews have studied the relationship between periodontitis and psoriasis (Qiao et al., 2019; Ungprasert et al., 2017). Ungprasert et al. reported a pooled risk ratio of 1.55 (95% CI, 1.35–1.77) for the presence of periodontitis in patients with psoriasis. When subgroup analysis was conducted according to study design, they estimated a higher risk among patients with periodontitis with a pooled RR of 2.33 (95% CI, 1.51–3.60) for case–control studies and a reduced pooled RR of 1.50 (95% CI, 1.37–1.64) for cohort studies. These trends of results are also observed in our review. But the authors did not evaluate the effect of periodontal diagnostic criteria on data synthesis. This could be a large concern, considering the significant heterogeneity for studies with insecure diagnosis methods showed in the present review. In another meta‐analysis, Qian et al. revealed that higher proportion of BOP, deeper PD, more CAL, fewer remaining teeth, and more missing teeth were observed in psoriasis group, indicating a worse periodontal status in patients with psoriasis than that of non‐psoriasis individuals (Qiao et al., 2019). They identified 8 publications, of which, 6 are case–control studies (Fadel et al., 2013; Mendes et al., 2019; Sharma et al., 2015; Skudutyte‐Rysstad et al., 2014; Ustun et al., 2013; Woeste et al., 2019) and two (Mayer, Elimelech, Balbir‐Gurman, Braun‐Moscovici, & Machtei, 2013; Sezer et al., 2016) are clinical trials. Not only patients with psoriasis but those with psoriatic arthritis were involved. Given that the variability in study designs and disease types among the original studies could dramatically increase the risk of selection bias, caution is thus required when interpreting their pooled results.

The present systematic review and meta‐analysis conducted strict inclusion and exclusion criteria. Data syntheses were based on studies with similar study design and diagnosis criteria to minimize the risk of bias. Although our results are limited by the small number of studies included, it is notable that the primary outcome indicates a statistically significant association between periodontitis and psoriasis. However, this review has several limitations. First, it builds only on case–control and retrospective cohort studies, no cause and effect relations could be established between periodontitis and psoriasis. Second, since only few studies reported periodontal clinical parameters, it was not possible to perform meta‐analyses for tooth loss, PD and CAL. Third, the risk of publication bias was not assessed due to the small number of included studies. Fourth, although there is the biological plausibility for a relationship between both diseases, the real association might be affected by other confounding factors like patients’ age, gender, or systemic conditions. Therefore, well‐designed prospective studies are needed to provide robust evidence for the association between the two conditions together with a restrictive diagnostic criterion for periodontitis and an appropriate confounding factors adjustment.

## 5 CONCLUSIONS

In conclusion, this systematic review with meta‐analysis reveals an association between periodontitis and psoriasis. However, this review was conducted with a relatively limited number of studies, which resulted in limitations for the pooled estimation of periodontal clinical parameters. Due to the relatively low quality of included studies, our review also highlights the necessity of more studies, particularly well‐designed prospective studies in different populations with follow‐up periods to confirm the evidence.

## AUTHOR CONTRIBUTIONS

**Xinze Zhang:** Formal analysis; Investigation; Writing‐original draft; Writing‐review & editing. **Hongqiu Gu:** Methodology; Software; Writing‐original draft; Writing‐review & editing. **Shang Xie:** Conceptualization; Supervision; Validation; Writing‐review & editing. **Yingying Su:** Conceptualization; Funding acquisition; Supervision; Validation; Writing‐review & editing.

### PEER REVIEW

The peer review history for this article is available at https://publons.com/publon/10.1111/odi.13617.
